# L-Ascorbic Acid Inhibits Breast Cancer Growth by Inducing IRE/JNK/CHOP-Related Endoplasmic Reticulum Stress-Mediated p62/SQSTM1 Accumulation in the Nucleus

**DOI:** 10.3390/nu12051351

**Published:** 2020-05-08

**Authors:** Youn Kyung Choi, Jung-Il Kang, Sanghoon Han, Young Ree Kim, Jaemin Jo, Yong Woo Kang, Do Ryeon Choo, Jin Won Hyun, Young Sang Koh, Eun-Sook Yoo, Hee-Kyoung Kang

**Affiliations:** 1 Department of Medicine, School of Medicine, Jeju National University 102 Jejudaehakno, Jeju 63243, Korea; choiyk@jejunu.ac.kr (Y.K.C.); jikang0024@jejunu.ac.kr (J.-I.K.); hanimh@jejunu.ac.kr (S.H.); namu8790@jejunu.ac.kr (Y.R.K.); jaemin2s@daum.net (J.J.); ky3204@gmail.com (Y.W.K.); kiper3012@nate.com (D.R.C.); jinwonh@jejunu.ac.kr (J.W.H.); yskoh7@jejunu.ac.kr (Y.S.K.); eunsyoo@jejunu.ac.kr (E.-S.Y.); 2 Jeju Research Center for Natural Medicine, Jeju National University; 102 Jejudaehakno, Jeju 63243, Korea

**Keywords:** L-ascorbic acid, breast cancer, autophagy, ER stress, p62/SQSTM1, *IRE*–*JNK*–*CHOP* signaling

## Abstract

Anticancer effects of L-ascorbic acid (Vitamin C, L-AA) have been reported in various types of cancers. L-AA intake reduces breast cancer recurrence and mortality; however, the role of L-AA in the treatment of breast cancer remains poorly understood. In this study, we investigated the effect and mechanism action of L-AA on breast cancer growth. L-AA inhibited the growth of breast cancer cells by inducing apoptotic cell death at the evaluated treatment concentrations without affecting normal cells. Moreover, L-AA induces autophagosome formation via regulation of mammalian target of rapamycin (*mTOR*), Beclin1, and autophagy-related genes (*ATGs*) and increased autophagic flux. Notably, we observed that L-AA increased p62/SQSTM1 (sequestosome 1) protein levels. Accumulation of p62 protein in cancer cells in response to stress has been reported, but its role in cancer regulation remains controversial. Here, we demonstrated that L-AA-induced p62 accumulation is related to L-AA-induced breast cancer growth inhibition. Furthermore, L-AA induced endoplasmic reticulum (ER) stress via the *IRE*–*JNK*–*CHOP* (inositol-requiring endonuclease–c-Jun N-terminal kinase–C/EBP homologous protein) signaling pathways, which increased the nuclear levels of p62/SQSTM1. These findings provide evidence that L-AA-induced ER stress could be crucial for p62 accumulation-dependent cell death, and L-AA can be useful in breast cancer treatment.

## 1. Introduction

L-ascorbic acid (Vitamin C, L-AA) is an essential micronutrient that functions as a cofactor in various enzymatic reactions [1,2]. It is a known anti-oxidant [3] and is necessary for collagen formation [4], absorption and metabolism of metal ions such as iron and copper [5], and synthesis of neurotransmitters [6]. As L-AA plays an important role in human physiology, research on the relationship between L-AA and disease is still ongoing. L-AA has been effective in the treatment of oral diseases [7], cardiovascular diseases [8], iron deficiency anemia [9], diabetes [10], age-related eye disease [11], Alzheimer’s disease [12], and viral infections [13]. Moreover, L-AA inhibits the growth and metastasis of various types of cancers, including melanoma [14], breast cancer [15], gastric cancer [16], colorectal cancer [17], pancreatic cancer [18], and leukemia [19]. Many studies have reported that the L-AA concentration in the plasma of cancer patients (10~30 μM) is lower than that of healthy controls (50~100 μM) [20,21,22,23,24]. In adults, the recommended daily dose of L-AA is about 75–90 mg in USA [25], but administration of high-dose L-AA (8 g/day) is known to be effective in preventing common cold [26]. However, high-dose L-AA is controversial as a chemotherapeutic agent in patients with cancer [27]. Several previous studies have reported that administration of a daily dose of 10 g L-AA has a beneficial effect in patients with cancer [28,29,30,31], while some have reported no overall relationship with L-AA intake [32,33,34]. Regarding this controversy, several reports have noted that the administration route of high-dose of L-AA (oral or intravenous injection) is crucial [35]. Intravenous injections of L-AA are maintained at high level in the blood [36,37]. Based on this, it has been reported that L-AA is more effective in intravenous injection than in oral administration in cancer [38]. Therefore, well-designed clinical studies and more basic investigations are needed to validate L-AA as an effective treatment for patients with cancer.

On the other hand, endoplasmic reticulum (ER) stress is reportedly associated with the pathogenesis of various diseases such as, neurodegenerative diseases, inflammatory diseases, metabolic diseases, stroke, heart diseases, pulmonary fibrosis, and cancers [39,40,41,42]. When ER stress occurs, the unfolded protein response (UPR) is induced to protect cells from the issue of protein folding in the ER through the activation of the intracellular signaling pathway [43]. Therefore, the regulation of ER stress and UPR has been widely used as a therapeutic target for various diseases. If severe and prolonged ER stress is maintained in cancer, ER stress-mediated UPR induces the death mechanism of cancer cells [44]. Accordingly, ER stress inducers are favored as potential anticancer agents [45,46,47].

Breast cancer is the most common cancer among women, with the second-highest mortality rate after lung cancer [48]. Therefore, to treat patients with breast cancer, it is essential to uncover drugs that have superior efficacy with fewer side effects. Many epidemiological studies have reported that L-AA intake reduces breast cancer recurrence and mortality [49,50,51,52,53,54]. However, there is still a lack of understanding regarding the role of L-AA in the treatment of breast cancer. In this study, we investigated the effect of L-AA on the growth of breast cancer cells through ER stress-mediated pathways.

## 2. Materials and Methods

### 2.1. Material

L-AA and hydrogen peroxide (H_2_O_2_) were purchased from Sigma-Aldrich (St. Louis, MO, USA).

### 2.2. Cell Lines and Cell Cultures

HCC-38 and SKBR3 breast cancer cells were purchased from the Korean Cell Line Bank (KCLB, Seoul, Korea). HCC-38 and SKBR3 cells were maintained in the Roswell Park Memorial Institute (RPMI) 1640-medium with 10% fetal bovine serum (FBS) and 1% antibiotics. African green monkey kidney (Vero) and rat intestinal epithelial (RIE) cells (kindly provided by Dr. Seong Gyu Ko at Kyung Hee University, Seoul, Korea) were cultured in Dulbecco’s modified Eagle medium (DMEM) supplemented with 10% FBS and 1% antibiotics at 37 ℃ in a humidified atmosphere under 5% CO_2_.

### 2.3. Trypan Blue Assay

Vero, RIE, HCC-38, and SKBR3 cells were seeded onto 60 mm plates for 24 h. Cells were treated with L-AA (50, 100, and 200 μM) for 48 h and then harvested using trypsin-EDTA. After the cells were harvested and suspended with phosphate-buffered saline (PBS), the cell suspension was mixed with 0.4% trypan blue solution (1:1) and incubated for 2 min. Viable (trypan blue dye-excluding) and dead (trypan blue dye-including) cells were counted using a hemocytometer chamber under the microscope.

### 2.4. Colony Formation Assay

HCC-38 and SKBR3 cells were seeded in 12-well plates at a density of 500 cells/well for 24 h, and then treated with L-AA (50, 100, and 200 μM). Every 3 days, the medium was replaced with fresh medium containing L-AA. After a 14 day treatment, the medium was discarded, and cell colonies were stained with crystal violet (0.1% in 20% methanol) for 2 h. After the colonies were washed with PBS, images were obtained to record the results. Crystal violet was extracted using 33% acetic acid and quantified by measuring the absorbance at 570 nm using the microplate reader (Bio Tek Instrument Inc., Winooski, VT, USA).

### 2.5. Reactive Oxygen Species (ROS) Measurement

HCC-38 and SKBR3 cells were seeded onto 60 mm plates for 24 h. The cells were treated with L-AA (50, 100, and 200 μM) for 24 h and then treated with 10 μM of 2′-7′-dichlorodihydrofluorescein diacetate (H_2_DCF-DA, Molecular Probes, Eugene, OR, USA) for another 1 h. The cells were harvested using trypsin-EDTA and centrifuged at 1000 rpm for 5 min and analyzed by LSRFortessa flow cytometry.

### 2.6. Apoptosis Analysis Assay

HCC-38 and SKBR3 cells were seeded onto 60 mm plates for 24 h and treated with L-AA (50, 100, and 200 μM) for 48 h. The cells were collected using trypsin-EDTA and centrifuged at 1000 rpm for 5 min. After discarding the cell medium, cells were suspended in the binding buffer (BD Bioscience, San Jose, CA, USA) and stained with annexin V-fluorescein isothiocyanate (FITC) in the dark for 15 min, followed by the 7-aminoactinomycin D (7AAD) reaction for 15 min. Annexin V and 7AAD-stained cells were detected using LSRFortessa flow cytometry (BD Bioscience, San Jose, CA, USA).

### 2.7. Western Blotting

HCC-38 and SKBR3 cells were seeded onto 60 mm plates and maintained for 24 h. Cells were treated with L-AA (200 μM) for various time points (0–6 or 24 h) and harvested. Whole-cell lysates were prepared using the PRO-PREP protein extraction solution (iNtRON Biotechnology, Seoul, Korea). Protein concentrations were measured using the Bio-Rad Protein assay dye (Bio-Rad, Hercules, CA, USA) according to the manufacturer’s instructions. An equal amount of protein of total lysate was subjected to 8%–12% sodium dodecyl sulfate-polyacrylamide gel electrophoresis and transferred onto polyvinylidene difluoride membranes. The membranes were blocked with 5% nonfat dry milk for 1 h and incubated with the relevant primary antibodies overnight at 4 ℃. The following are the primary antibodies: anti-caspase-12, C/EBP homologous protein (*CHOP*), and p62/SQSTM1 (sequestosome 1) antibodies were purchased from Abcam (Cambridge, MA, USA); anti-*ATF4* (activating transcription factor 4), -*elF2α* (eukaryotic initiation factor 2α), -p-*elF2α*, and -p-*IRE1α* were obtained from GeneTex Inc. (Irvine, CA, USA); anti-β-actin and -*Bcl2* (B-cell lymphoma 2) were purchased from Santa Cruz Biotechnology (Santa, CA, USA); anti-p-PERK was purchased from MyBioSource (San Diego, CA, USA); anti-*ATG3*, -*ATG7*, -Beclin1, -*Bip*, - *IRE1α*, - *LC3*Ⅰ/Ⅱ, -*PERK*, -p-*JNK*, and -p-*mTOR* were obtained from Cell Signaling (Danvers, MA, USA). After washing, the membranes were incubated with horseradish peroxidase-labeled anti-rabbit IgG or -mouse IgG secondary antibodies at room temperature for 1 h. Immunoreactive protein was exposed to X-ray films using West-zol^TM^ Plus reagents (iNtRON Biotechnology, Seoul, Korea).

### 2.8. Immunocytochemistry

HCC-38 cells were seeded in 6-well plates with cover-glasses for 24 h, and then treated with L-AA (200 μM) for 6 h. The cells were fixed with 4% paraformaldehyde for 10 min and washed with PBS three times. For permeabilization, the cells were incubated with 0.5% Triton X-100 for 7 min and washed. The cells were blocked with blocking buffer (10% FBS and 1% bovine serum albumin in 0.1% Tween-20 buffer) for 2 h, and then stained with anti-p62/SQSTM1 primary antibody (1 μg/mL) and anti-Alexa Fluor-594 antibody (Invitrogen, Carlsbad, CA, USA 1:200) for 1 h at room temperature in the dark. After washing, the cells were mounted with 4′,6-diamidino-2-phenylindole (DAPI)-contained VECTASHIELD mounting medium (Vector Laboratories, Burlingame, CA, USA). Images were acquired using a confocal microscope (FV10i, Olympus, Melville, NY, USA).

### 2.9. Surface DR5 Expression Analysis

HCC-38 cells were seeded onto 60 mm plates and treated with L-AA (50, 100, and 200 μM) for 24 h. The cells were treated with trypsin-EDTA and centrifuged at 1000 rpm for 5 min. After washing with PBS, the cells were incubated with anti-death receptor 5 (*DR5*) primary antibody (Abcam, Cambridge, MA, USA, 1:100) in blocking buffer (2% FBS in PBS) for 30 min on ice. The cells were washed and stained with anti-Alexa Fluor-488 secondary antibody (Invitrogen, Carlsbad, CA, 1:1000) or rabbit IgG monoclonal isotype control (Abcam, Cambridge, MA, USA) in the dark for 30 min on ice. DR5-positive cells were detected using LSRFortessa flow cytometry (BD Bioscience, San Jose, CA, USA).

### 2.10. Transfection

For *CHOP* or p62 transient knockdown, HCC-38 cells were seeded onto 6-well plates and transfected with siRNA (Bioneer, Daejeon, Korea) using the Lipofectamine reagent (Invitrogen, Carlsbad, CA, USA) according to the manufacturer’s instruction. After the transfected cells were treated with L-AA (200 μM) for 6 h, western blotting, the trypan blue assay, colony formation assay, and immunocytochemical experiments were performed.

### 2.11. Inhibitor Treatment

HCC-38 cells were seeded and pre-treated with ER stress inhibitors (4μ8C: inositol-requiring endonuclease 1α (*IRE1α*) inhibitor, 10 μM; SP600125: c-Jun N-terminal kinase (*JNK*) inhibitor, 1 μM; GSK2606414: PKR-like ER kinase (*PERK*) inhibitor, 10 μM; Salubrinal: *elF2α* inhibitor, 10 μM) for 1 h, and then treated with L-AA (200 μM) for 6 h. Thereafter, trypan blue assay, western blotting, and immunocytochemical experiments were performed. SP600125, 4μ8C, GSK2606414, and Salubrinal were purchased from Calbiochem (Cambridge, MA, USA).

### 2.12. Statistical Analysis

Results are shown as means ± standard deviation (SD) from three independent experiments. A *p*-value less than 0.05 in the two-tailed Student’s *t*-test was considered significant.

## 3. Results

### 3.1. L-AA Induces Apoptosis of Breast Cancer Cells

**Figure 1:**
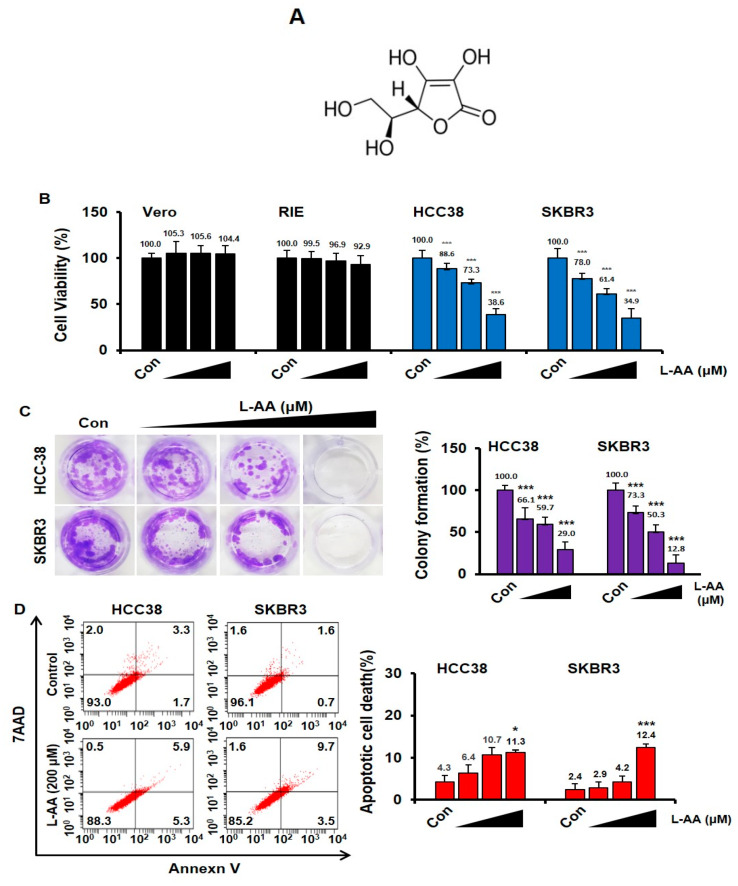
L-ascorbic acid (L-AA) inhibits breast cancer growth. (**A**) The chemical structure of L-AA. (**B**) Normal cells (Vero and rat intestinal epithelium (RIE)) and breast cancer cells (HCC38 and SKBR3) were treated with various concentrations of L-AA (50, 100, and 200 μM) for 48 h and then stained with trypan blue. Viable and dead cells were counted. (**C**) Breast cancer cells were treated with L-AA (50, 100, and 200 μM) every 3 days. After the 14 day treatment, cell colonies were stained with crystal violet. Crystal violet stained cells were extracted using 33% acetic acid and quantified by measuring absorbance at 570 nm on the microplate reader. (**D**) Breast cancer cells were treated with L-AA (50, 100, and 200 μM) for 48 h and then stained with annexin V-FITC and 7AAD in the binding buffer at room temperature in the dark. Stained cells were detected by LSRFortessa flow cytometry. The graph shows the sum of annexin V-FITC alone-positive cells (early apoptotic cells) and annexin V-FITC and 7AAD double positive cells (late apoptotic cells) from whole stained cells. Data are shown as the mean of three independent experiments, and the error bars represent standard deviation (SD). * *p* < 0.05 and *** *p* < 0.001 versus the control group.

Breast cancer (HCC38 and SKBR3) and normal cells (Vero and RIE) were treated with L-AA at various concentrations (50, 100, and 200 μM) for 48 h. The viability of Vero and RIE cells was not affected, whereas the viability of breast cancer cells was reduced by L-AA treatment (Figure 1B). To confirm the long-term effect of L-AA treatment, we performed colony formation experiments. When HCC38 and SKBR3 were treated with L-AA for 14 days, L-AA decreased colony formation in a dose-dependent manner when compared with that in the control group (Figure 1C). To determine whether L-AA suppresses cell viability by inducing apoptosis, we performed annexin V and 7AAD staining. L-AA increased the percentage of annexin V-positive apoptotic cells (Figure 1D). These results suggest that L-AA suppresses the growth of breast cancer cells by inducing apoptotic cell death.

### 3.2. L-AA-Induced Apoptosis Is Not Correlated to the Intracellular ROS Generation

**Figure 2:**
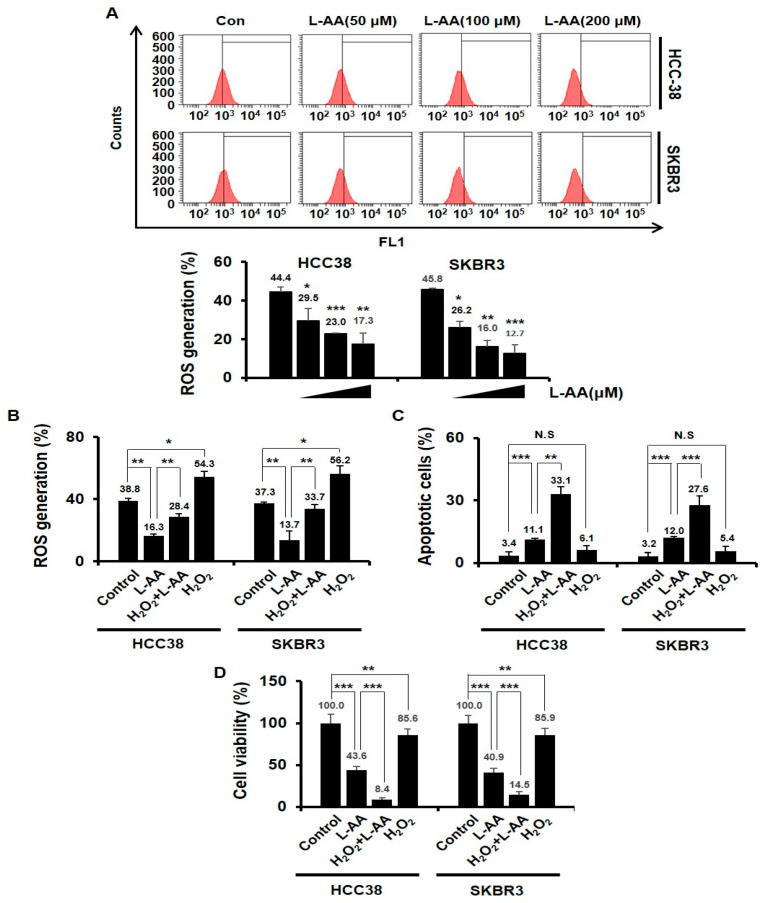
L-AA-induced apoptosis is not correlated to the intracellular reactive oxygen species (ROS) generation. (**A**) Breast cancer cells (HCC38 and SKBR3) were treated with various concentrations of L-AA (50, 100, and 200 μM) for 24 h and then treated with H_2_DCF-DA fluorescent dye for 1 h at 37 °C. ROS generation was measured by LSRFortessa flow cytometry. Graph shows the proportion of H_2_DCF-DA-positive cells in the total cells. (**B**) Breast cancer cells were pre-treated with L-AA (200 μM) for 1 h, followed by exposure to H_2_O_2_ (10 μM) for 24 h. ROS production was measured. (**C**,**D**) HCC38 and SKBR3 cells were pre-treated with L-AA (200 μM) followed by exposure to H_2_O_2_ (10 μM) for 48 h. (**C**) Stained with annexin V-FITC and 7AAD and analyzed by LSRFortessa flow cytometry. The graph shows the proportion of annexin V-FITC alone-positive cells (early apoptotic cells) and annexin V-FITC and 7AAD double positive cells (late apoptotic cells) in whole stained cells. (**D**) Trypan blue assay was performed. Data are shown as the mean of three independent experiments, and the error bars represent standard deviation (SD). * *p* < 0.05, ** *p* < 0.01, and *** *p* < 0.001 were considered statistically significant.

Whether L-AA acts an anti-oxidant or pro-oxidant in cancer is still controversial [55,56,57,58]. We thus examined L-AA-induced ROS production. When HCC38 and SKBR3 breast cancer cells were treated with L-AA for 24 h, L-AA decreased ROS production in a dose-dependent manner when compared with that in the control group (Figure 2A). To determine whether the decrease of ROS production by L-AA is required for L-AA-induced breast cancer cell death, HCC38 and SKBR3 breast cancer cells were treated with hydrogen peroxide (H_2_O_2_), one of the pro-oxidants. The H_2_O_2_ treatment increased the ROS production when compared with that in the control group, while the H_2_O_2_ treatment in combination with L-AA attenuated the decrease of intracellular ROS levels by L-AA (Figure 2B). The H_2_O_2_ treatment in combination with L-AA increased apoptotic cell death and decreased cell viability when compared with L-AA treatment alone in breast cancer cells (Figure 2C,D). H_2_O_2_ treatment alone reduced the viability of breast cancer cells when compared with that in the control group, but it was not related to apoptosis (Figure 2C,D). These results indicate that L-AA-induced apoptosis of breast cancer cells was not correlated to the intracellular ROS generation.

### 3.3. L-AA Induces Autophagosome Formation, While Increasing p62 Accumulation in the Nucleus

**Figure 3:**
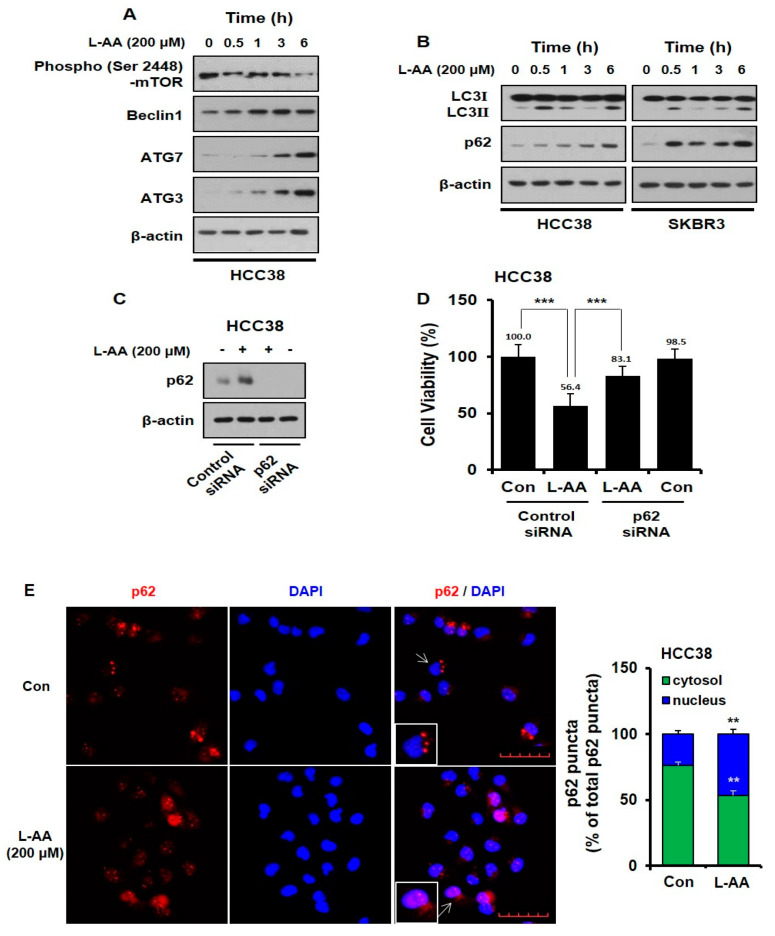
L-AA induces autophagosome formation, while L-AA increases p62 accumulation in the nucleus. (**A,B**) Breast cancer cells were treated with L-AA (200 μM) for indicated hours (0–6 h) and then western blotting was performed with anti-p-*mTOR*, -Beclin1, -*ATG*7, -*ATG*3, -*LC3*Ⅰ/Ⅱ, and -p62. β-actin was used as the loading control. (**C,D**) HCC-38 cells were transfected with p62 siRNA and treated with 200 μM of L-AA. (**C**) Western blotting was performed with anti-p62 and β-actin was used as the internal control. (**D**) Trypan blue assay was performed. (**E**) HCC-38 cells were treated with 200 μM of L-AA for 6 h. Cells were stained with anti-p62 (1 μg/mL) and –AlexaFluor-594 secondary antibody (1:200). Images were obtained using the FV10i confocal microscope, using the 40× objective; the scale bar indicates 50 μm. When the experiment was performed, three or more fields were obtained for each group, and p62 puncta were counted and averaged. This process was repeated three times, and the mean value of three experiments was statistically processed. Data are shown as the mean of three independent experiments, and the error bars represent standard deviation (SD). ** *p* < 0.01 and *** *p* < 0.001 were considered statistically significant.

To identify the mechanism by which L-AA induces apoptosis, we examined autophagy. Mammalian target of rapamycin (*mTOR*) and Beclin1 are two important molecules involved in autophagy initiation and autophagosome formation [59]. Microtubule-associated protein 1A/1B-light chain 3 (*LC3*)Ⅰ is combined with phosphatidylethanolamine (PE) to form *LC3*Ⅱ via autophagy-related gene (*ATG*)-7 and −3 [60]. L-AA decreased *mTOR* phosphorylation in a time-dependent manner, while the level of Beclin1 was increased (Figure 3A). Furthermore, L-AA increased the expression of *ATG*-7 and *ATG-3* (Figure 3A). L-AA also increased the expression of *LC3*Ⅱ, which means that the number of autophagosomes increased (Figure 3B). Sequestosome 1 (p62/SQSTM1) is inserted into autophagosomes and degrades through the formation of an autolysosome fused with an autophagosome and lysosome. Thus, p62 has been known as an another autophagy marker degraded by autophagy induction [61]. However, we observed that p62 protein levels were increased by L-AA, although autophagic flux increased (Figure 3B). 

In contrast, recent studies have reported that p62 expression can be upregulated by stress conditions, including oxidative stress, starvation, and accumulation of dysfunctional proteins [62,63,64]. Thus, we assessed whether L-AA could induce stress-mediated autophagy and p62 accumulation. To investigate the role of p62 on L-AA-inhibited cell viability, HCC38 cells were transfected with p62 siRNA. As shown in Figure 3C,D, following L-AA treatment, p62 silencing significantly increased cell viability when compared with the control siRNA-transfected cells. Recent reports suggest that DNA damage-induced by the inhibition of DNA repair requires p62 accumulation in the nucleus, which is associated with cell death [65,66]. As shown in Figure 3E, compared with control cells in which p62 is in the cytoplasm, the level of p62 increased in the nucleus of L-AA-treated cells. This indicates that L-AA induces stress-mediated autophagy and cell death via the accumulation of p62 in the nucleus.

### 3.4. L-AA Induces ER Stress in Breast Cancer Cells

**Figure 4:**
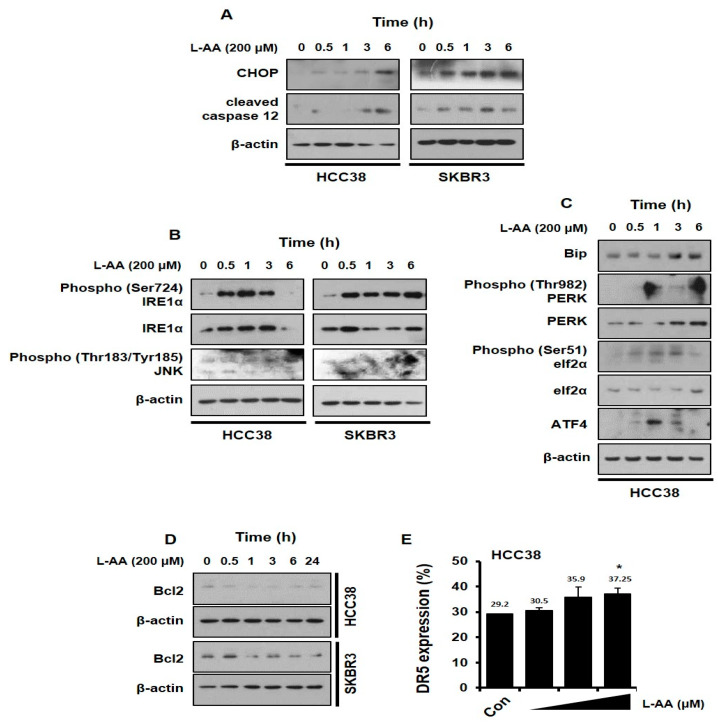
L-AA increases endoplasmic reticulum (ER) stress in breast cancer cells. (**A**,**D**) HCC-38 or SKBR3 cells were treated with 200 μM of L-AA for indicated hours, and then western blotting was performed. β-actin was used as the loading control. (**A**) Expression levels of C/EBP homologous protein (*CHOP*) and cleaved caspase 12. (**B**) Protein levels involved in ER stress associated inositol-requiring endonuclease 1 (*IRE1*) signaling pathway. (**C**) Protein levels involved in ER stress-related PKR-like ER kinase (*PERK*) signaling pathway. (**D**) B-cell lymphoma 2 (*Bcl2*) expression levels (**E**) HCC-38 cells were treated with 200 μM of L-AA for 24 h and stained with anti-*DR5* (death receptor 5) primary antibody (1:100) and –AlexaFluor-488 secondary antibody (1:1000). *DR5*-positive cells were detected by LSRFortessa flow cytometry. Data are shown as the mean of three independent experiments, and the error bars represent standard deviation (SD). * *p* < 0.05 versus the control group.

A recent report indicates that ER stress is associated with the apoptotic pathway and p62 accumulation [67,68]. To investigate whether L-AA induces ER stress, we evaluated the expression of ER stress markers including C/EBP homologous protein (*CHOP*) and cleaved caspase 12. Breast cancer cells were treated with L-AA and western blotting was performed to confirm the *CHOP* and cleaved caspase 12 expression. L-AA enhanced *CHOP* and cleaved caspase 12 levels in HCC38 and SKBR3 cells (Figure 4A). Next, we examined the effect of L-AA on ER stress-related pathways regulating *CHOP*, including inositol-requiring endonuclease 1 (*IRE1α*) and PKR-like ER kinase (*PERK*) signaling. The two pathways including IRE–c-Jun N-terminal kinase (*JNK*) and PERK–eukaryotic initiation factor 2 (*elF2*)-α are crucial for the transmission of ER-stress signals to *CHOP* in cancer [69,70]. L-AA induced the phosphorylation of *IRE1α* and *JNK* levels (Figure 4B). Moreover, L-AA increased binding immunoglobulin protein (*Bip*), p-*PERK*, p-*elF2α*, and activating transcription factor 4 (*ATF4*), indicating the activation of the *PERK* signaling pathway (Figure 4C). As a transcription factor, *CHOP* regulates the expression of a variety of apoptosis-related genes, including B-cell lymphoma 2 (*Bcl2*) and *DR5* [71,72]. L-AA decreased *Bcl2* levels and increased membrane *DR5* expression (Figure 4D,E). Therefore, the results indicate that L-AA induces ER stress in breast cancer cells.

### 3.5. L-AA Inhibits Breast Cancer Growth via IRE1/JNK/CHOP Signaling

**Figure 5:**
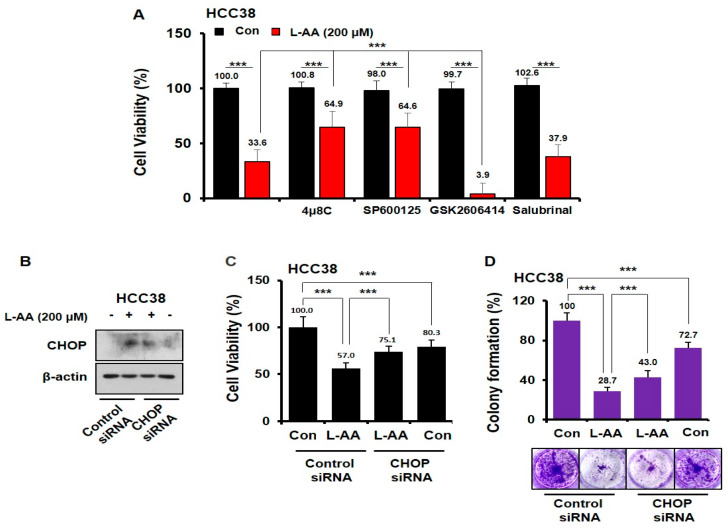
L-AA enhances *IRE1*– c-Jun N-terminal kinase (*JNK*)–*CHOP*-mediated cell death. (**A**) HCC-38 cells were pre-treated with ER stress inhibitors (10 μM of 4μ8C, 1 μM of SP600125, 10 μM of GSK2606414, and 10 μM of Salubrinal) for 1 h, and then treated with L-AA (200 μM) for 48 h. Cell viability was measured using the trypan blue assay. (**B**,**D**) HCC-38 cells were transfected with *CHOP* siRNA. (**B**) *CHOP* siRNA-transfected cells were treated with 200 μM of L-AA for 6 h and then western blotting was performed (**C**) *CHOP* siRNA-transfected cells were treated with 200 μM of L-AA for 48 h, followed by the trypan blue assay (**D**) *CHOP* siRNA-transfected cells were treated with 200 μM of L-AA for 10 days, followed by the colony formation assay. Data are shown as the mean of three independent experiments, and the error bars represent standard deviation (SD). *** *p* < 0.001 was considered statistically significant.

To confirm the role of the *IRE*–*JNK* and *PERK*–*elF2α* pathways in L-AA-induced cell death, experiments using ER stress inhibitors were performed. We observed that GSK2606414 (*PERK* inhibitor) or Salubrinal (*elF2α* inhibitor) in combination with L-AA did not restore growth inhibition induced by L-AA (Figure 5A). Interestingly, 4μ8C (*IRE1α* inhibitor) + L-AA treatment or SP600125 (*JNK* inhibitor) + L-AA treatment increased cell viability when compared with L-AA treatment alone (Figure 5A). None of the inhibitors affected cell viability (Figure 5A). In addition, L-AA increased the cell viability and colony formation in *CHOP*-knockdown cells when compared with those in control siRNA-treated cells (Figure 5B–D). However, cytotoxicity was observed only in *CHOP*-knockdown cells (Figure 5C,D). Thus, our data suggest that L-AA inhibited the growth of breast cancer cells via activation of the *IRE* signaling pathway.

### 3.6. L-AA-Induced IRE Signaling Causes p62 Accumulation in the Nucleus

**Figure 6:**
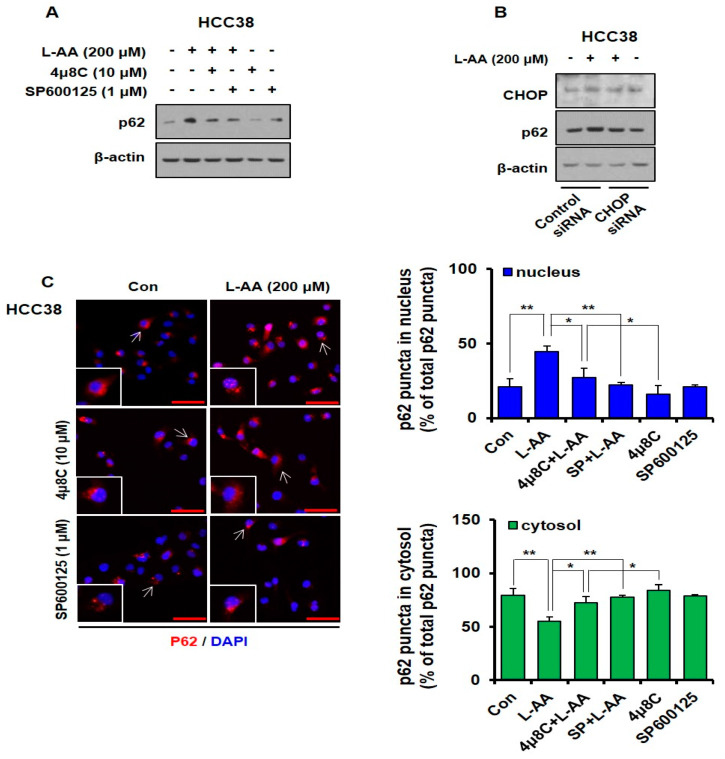
L-AA-induced *IRE1*–*JNK*–*CHOP* activation causes p62 accumulation in the nucleus. (**A**) HCC-38 cells were pre-treated with *IRE1* signaling pathway inhibitors such as 4μ8C (10 μM) and SP600125 (1 μM) for 1 h and treated with L-AA (200 μM) for 6 h. Western blotting was performed with anti-p62, and β-actin was used as the internal control. (**B**) HCC-38 cells were transfected with *CHOP* siRNA, treated with L-AA (200 μM) for 6 h, and then western blotting was performed. (**C**) HCC-38 cells were pre-treated with the *IRE1* signal pathway inhibitors, such as 4μ8C (10 μM) and SP600125 (1 μM), and treated with L-AA (200 μM) for 6 h. Cells were stained with anti-p62 (1 μg/mL) and –AlexaFluor-594 secondary antibody (1:200). Images were obtained using the FV10i confocal microscope, using the 40× objective; the scale bar indicates 50 μm. Following the experiment, three or more fields were obtained for each group, and p62 puncta were counted and averaged. This process was repeated three times, and the mean value of three experiments was statistically processed. The following graph shows the ratio of p62 puncta expressed in the cytosol and nucleus, based on 100% of total p62 puncta. Data are shown as the mean of three independent experiments, and the error bars represent standard deviation (SD). * *p* < 0.05 and ** *p* < 0.01 were considered statistically significant.

To further confirm whether the activation of the *IRE*/*JNK*/*CHOP* signal regulates L-AA-induced p62 expression and accumulation in the nucleus, inhibitor and knockdown experiments were performed. Inhibitors of the IRE pathway, 4μ8C (*IRE1α* inhibitor) and SP600125 (*JNK* inhibitor), decreased L-AA-induced p62 expression levels. The treatment with 4μ8C or SP600125 did not affect the p62 expression levels (Figure 6A). In HCC38 cells, knockdown of *CHOP* attenuated L-AA-increased p62 levels when compared with the control siRNA + L-AA group (Figure 6B). Next, confocal experiments were performed to confirm the localization of p62 using combination treatment with L-AA and *IRE* signal inhibitors. L-AA alone increased the nuclear level of p62, while co-treatment with L-AA and inhibitors (4μ8C and SP600125) attenuated L-AA-induced nuclear accumulation of p62 (Figure 6C). Our findings suggest that inhibition of the *IRE* pathway suppresses the nuclear accumulation of p62 induced by L-AA in breast cancer cells.

## 4. Discussion

Although the anticancer effect of L-AA has been demonstrated in various experiments, it is still controversial to administer L-AA in patients with cancer due to a lack of mechanism-based studies. In the present study, we demonstrated that L-AA induced apoptotic death and ER stress in breast cancer cells. Furthermore, we observed that L-AA increased the nuclear level of p62 via ER stress-mediated *IRE*–*JNK*–*CHOP* signaling pathways.

L-AA acts as an anti-oxidant or as pro-oxidant in cancer, so it is still controversial [55,56,57,58]. Unlike some previous studies that ascorbate is oxidized in the medium and consequently ROS is generated, our study showed that L-AA decreased intracellular ROS levels (Figure 2A). Furthermore, when treated with H_2_O_2_ acting as a pro-oxidant, it was observed that the decrease of ROS by L-AA was not correlated with apoptosis induction of breast cancer cells by L-AA (Figure 2C,D). The oxidative state of L-AA is crucial to the anticancer effects of L-AA in many studies, but our study showed that the anticancer effect of L-AA in breast cancer cells was independent of the oxidative state of L-AA. During carcinogenesis, the accumulation of aberrant proteins in the ER by chromosomal rearrangements, hypoxia, and environment factors induces ER stress [73,74,75]. Cells minimize ER stress by activating the UPR system to protect themselves and maintain homeostasis during ER stress [43,44]. The UPR system is mediated by *IRE*, *PERK*, and *ATF6*, which are located on the ER membrane and activated by ER stress stimulation [76,77]. UPR increases the expression of chaperone proteins, improving the function of ER in terms of protein synthesis and folding. Additionally, UPR decreases the amount of protein that enters the ER or increases the degradation of unfolded proteins in the ER, thereby reducing the ER burden and consequently suppressing ER stress [78,79]. Nevertheless, if the ER stress is not reduced and the ER fails to restore its function, apoptosis is activated to remove damaged cells [79]. Therefore, the mechanism of ER stress-mediated apoptosis could be utilized as a new therapeutic target for cancer research. Various compounds regulating this mechanism are being studied and validated [47,80]. Transcription of the *CHOP* gene is extremely crucial in ER stress-mediated apoptosis, which is activated by *IRE1*, *PERK*, and *ATF6* [81,82]. ER stress-mediated apoptosis is less induced in *CHOP*-deficient cells, whereas overexpression of the *CHOP* gene promotes apoptosis [83,84]. Furthermore, *CHOP* also reduces *Bcl2* and upregulates *DR5*, followed by the induction of the apoptosis pathway [62]. 

ER stress-mediated apoptosis can also be induced by *IRE1*-mediated *JNK* activity and caspase 12, an ER stress-specific caspase located on the outer layer of the ER [79]. In this study, L-AA induced the activation of two important signaling pathways in ER stress, *IRE1*–*JNK* and *PERK*–*elF2α*–*ATF4* (Figure 4B,C). However, the *PERK*–*elF2α*–*ATF4* pathway was not associated with the anticancer effect of L-AA, and the inhibition of L-AA-mediated cell viability was only improved by *IRE1*–*JNK* signaling (Figure 5A). In addition, L-AA increased the expression of ER stress-mediated apoptosis markers, cleaved caspase 12 and *CHOP*, and the increased *CHOP* was crucial for L-AA-inhibited cell growth (Figure 4A and Figure 5B–D). Moreover, the expression of *Bcl2* and *DR5*, which are important for apoptosis induction controlled by *CHOP* transcriptional activity, was regulated by L-AA (Figure 4D,E). *CHOP* is known to be a potential target for drug development in cancer [85]. Reportedly, resveratrol [86], Polyphenon E^®^ [87], gartanin [88], garcinol [89], and *Clinacanthus nutans* [90] exhibit anticancer effects via *CHOP* regulation.

ER stress induces processes related to cell survival and death, such as autophagy [91,92,93]. Autophagy is an important process for maintaining homeostasis in normal cells and it is a cellular degradation pathway for the removal of damaged or superfluous proteins and organelles [94]. During autophagy, cytoplasmic materials are sequestered by the autophagosome, a double-membrane structure, and fused with the lysosome for degradation [95]. Autophagy is valuable as a therapeutic target for cancer treatment since it has been reported that it regulates initiation, growth, survival, malignancy, and metastasis of cancer [96,97]. *mTOR* is a major negative regulator of autophagy and is important in regulating the activity of kinases, including UNC-51-like kinase 1 (*ULK1*) [98,99]. Beclin1 induces autophagy by forming the Beclin1–class III phosphatidylinositol 3-kinase (*Vps34*) complex. Additionally, the SH3 domain of Beclin1 binds to anti-apoptotic *Bcl2* to regulate apoptosis [100]. In our study, L-AA increased the initiation of autophagy and autophagosome formation, which are regulated by the activation of *mTOR* and the increase of Beclin1, *ATG*-7, and -3 (Figure 3A). As a result, the conversion of *LC3*Ⅱ was enhanced by L-AA (Figure 3B). As a chaperone of ubiquitinated proteins of the autophagosome, p62/SQSTM1, binds to *LC3* of the autophagosome and is itself degraded by autophagy [101]. 

However, we unexpectedly observed a significant increase in the p62 protein levels in breast cancer cells treated with L-AA (Figure 3B). Additionally, knockdown of p62 expression attenuated L-AA-inhibited cell viability (Figure 3D). Consistent with our results, several studies have shown that autophagic cell death through p62 increasingly inhibits cancer growth in hepatocellular carcinoma [68,102], leukemia [103], breast cancer [104], and pleural mesothelioma cells [105]. Moreover, p62 upregulation has been observed when ER stress-mediated apoptosis occurred, which is related to the ER stress-activated *IRE1*–*JNK* signaling pathway [68,105]. Our study also confirmed that L-AA increased p62 levels through *IRE*–*JNK*–*CHOP* pathway (Figure 6A,B). Furthermore, recent studies have shown that ER stress increases the accumulation of p62 by blocking autophagosome–lysosome fusion and inhibiting lysosomal functions [106]. p62 is a nucleocytoplasmic shuttling protein, the nuclear roles of which remains largely unknown. Recently, p62 accumulation in the nucleus has been reported to induce DNA damage-associated cell death [65,66]. In addition, low nuclear p62 expression is related to a high histologic grade, as well as poor overall and disease-specific survival in oral squamous cell carcinoma [107]. In this study, L-AA increased nuclear p62, which correlated with the ER stress-mediated *IRE*–*JNK* signaling pathway (Figure 3E and Figure 6C).

Breast cancer is a heterogeneous disease and is classified into different subtypes depending on the presence or absence of hormone receptors such as estrogen receptor (*EsR*), progesterone receptor (*PR*), and human epidermal growth factor receptor 2 (*HER2*). Unlike other cancers, hormone therapy and *HER2*-targeted therapy are possible depending on the characteristics and subtype of the patients with breast cancer [108,109]. Because heterogeneity of breast cancer subtypes is the most common cause of therapy failure, it is important to proceed with research or development of therapeutic agents considering breast cancer subtypes [110]. On the other hand, the sensitivity of L-AA in breast cancer cells is known to correlate with the expression of sodium-dependent vitamin C transporter-2 (*SVCT*-*2*), which transports L-AA to cells [58]. *SVCT-2* expression is revealed to be higher in *EsR*/*PR*-negative than in *EsR*/*PR*-positive breast cancer patient tissues [58]. The SKBR3 cell line used in our study was reported to have high *SVCT-2* expression and high sensitivity to L-AA treatment as an *ER*/*PR*-negative cell line [58]. HCC38 cells are also known as *EsR*/*PR*-negative breast cancer cell line [111], but the expression of *SVCT-2* in HCC38 cells has not been reported. The amount of ascorbate in cancer cells which is dependent on the expression of *SVCT-2*, is crucial to the efficacy of L-AA in clinical and in vivo studies. To further verify the efficacy of L-AA in breast cancer, we will investigate the expression and regulatory mechanisms of *SVCT-2* that determine the sensitivity of L-AA and conduct in vivo studies using xenograft animal model in further research.

## 5. Conclusions

In conclusion, we observed that L-AA induces p62 accumulation in the nucleus through the ER stress-mediated *IRE1*–*JNK*–*CHOP* pathway, thereby promoting cell death. Our study provides scientific evidence regarding the applicability of L-AA treatment in breast cancer patients.
